# Cardiovascular Autonomic Dysfunction in Cancer Therapy: Clinical Manifestations and Management

**DOI:** 10.14740/cr2242

**Published:** 2026-07-17

**Authors:** Tyler Abram Kilgore, Sruthi Vellanki, Sravya Vellanki, Jeffrey Curran Henson, Srikanth Vallurupalli

**Affiliations:** a Department of Internal Medicine, University of Arkansas for Medical Sciences, Little Rock, AR, USA; b Division of Hematology and Medical Oncology, University of Arkansas for Medical Sciences, Little Rock, AR, USA; c Department of Internal Medicine, Willis Knighton Health, Shreveport, LA, USA; d Division of Cardiology, University of Arkansas for Medical Sciences, Little Rock, AR, USA

**Keywords:** Cardiovascular autonomic dysfunction, Cardio-oncology, Heart rate variability, Orthostatic hypotension, Sinus tachycardia, Mindfulness, Exercise, Yoga

## Abstract

Cardiovascular autonomic dysfunction is an underrecognized manifestation of cancer therapy–related cardiotoxicity with important clinical implications. Disruption of autonomic regulation during chemotherapy most commonly manifests as orthostatic hypotension, inappropriate sinus tachycardia, and reduced heart rate variability, often occurring in the absence of overt structural heart disease. These abnormalities may contribute to symptom burden, impaired functional capacity, and increased cardiovascular risk in patients undergoing cancer treatment. Emerging data indicate that autonomic dysfunction in this setting may be modifiable. Among available interventions, structured exercise programs demonstrate the strongest and most consistent improvements in autonomic indices, alongside established benefits in functional capacity, fatigue, and quality of life. Relaxation and mindfulness-based interventions, including yoga, offer low-risk adjunctive benefits, whereas cryotherapy remains exploratory. Pharmacologic therapies play a symptom-directed role, particularly for orthostatic hypotension, but are often constrained by comorbid cardiovascular disease and limited oncology-specific evidence. This review evaluates current evidence on the clinical manifestations and therapeutic strategies for chemotherapy-associated cardiovascular autonomic dysfunction, highlighting opportunities to improve patient-centered outcomes in cardio-oncology care.

## Introduction

**Figure 1:**
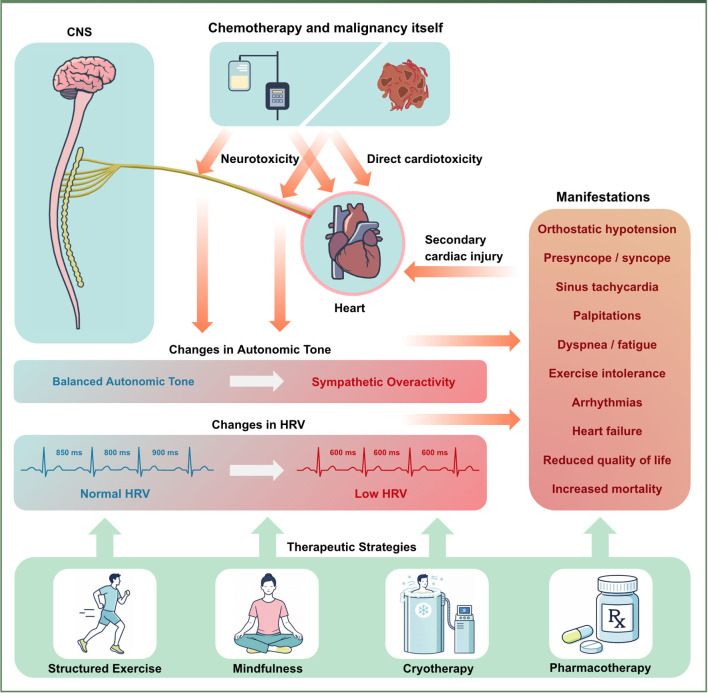
Overview of mechanisms, clinical manifestations, and therapeutic targets in chemotherapy-associated cardiovascular autonomic dysfunction. Chemotherapy and malignancy can affect the central and peripheral autonomic nervous system through neurotoxicity and direct cardiotoxic effects, resulting in altered autonomic tone with sympathetic overactivity and reduced heart rate variability (HRV). These changes contribute to clinical manifestations including orthostatic hypotension, sinus tachycardia, palpitations, exercise intolerance, arrhythmias, and heart failure. Potential therapeutic strategies aimed at improving autonomic balance include structured exercise, mindfulness-based interventions, cryotherapy, and pharmacologic therapy.

Chemotherapy has revolutionized cancer treatment, extending survival and improving quality of life for countless patients. However, the wide range of toxicities associated with chemotherapeutic agents remains a major challenge in modern oncology [1]. While the direct cardiotoxic effects of chemotherapy, such as cardiomyopathy and heart failure, have been extensively studied, substantially less attention has been given to the impact of chemotherapy on cardiovascular autonomic regulation. Recent European Society of Cardiology (ESC) guidelines on cardio-oncology emphasize the broader spectrum of cancer therapy–related cardiovascular toxicity, yet autonomic dysfunction remains largely unaddressed [2]. The autonomic nervous system (ANS) serves as a central regulator of unconscious cardiovascular function through coordinated sympathetic and parasympathetic activity. Disruption of this balance may result in clinically meaningful cardiovascular consequences [3]. Although the precise mechanisms underlying chemotherapy-associated cardiovascular autonomic dysfunction remain incompletely defined, available evidence suggests a multifactorial process involving neural injury and inflammatory signaling. Experimental and clinical studies indicate that chemotherapeutic agents may disrupt autonomic regulation through oxidative stress, mitochondrial dysfunction, neuroinflammation, and direct neuronal toxicity, mechanisms well described in chemotherapy-induced peripheral neuropathy and likely shared by autonomic neurons [4]. These processes may impair both sympathetic and parasympathetic signaling and provide a biologic framework for the clinical manifestations observed in affected patients. This review evaluates current evidence on cardiovascular autonomic dysfunction associated with cancer therapy, with particular emphasis on chemotherapy-associated autonomic dysfunction, where the evidence base is most developed. The primary objective is to review the clinical manifestations, evaluation, and management of cardiovascular autonomic dysfunction in patients receiving cancer-directed therapy. The secondary objective is to summarize proposed mechanisms, discuss the strength and limitations of the available evidence, and distinguish oncology-specific data from selected evidence extrapolated from non-oncology populations when direct oncology evidence is limited. Prior work by Yoon and Oh [5] has helped establish recognition of cardiovascular autonomic dysfunction before and after chemotherapy; the present review builds on that foundation with a more clinician-oriented focus on diagnosis, established management strategies, emerging interventions, and referral considerations. The proposed mechanisms, key manifestations, and therapeutic approaches are summarized in Figure 1. Representative clinical manifestations and associated therapeutic strategies reported in the literature are summarized in Table 1.

**Table 1:** Selected Studies Evaluating Manifestations and Interventions in Chemotherapy-Associated Cardiovascular Autonomic Dysfunction

Ref. No.	First author	Population studied	n	Study design	Chemotherapy	Intervention	Primary findings / conclusion
6	Stone	Advanced cancer patients (palliative care cohort)	185	Observational, prospective cohort	Not specified	None	Cardiovascular autonomic dysfunction highly prevalent; up to ∼80% demonstrated definite/severe dysfunction. Associated with fatigue and reduced survival.
7	Iwański	Broad oncology patients	223	Observational, cross-sectional	Not specified	None	Cancer patients receiving chemotherapy demonstrated clinically significant orthostatic hypotension consistent with autonomic dysfunction.
8	Dermitzakis	Ovarian cancer patients	31	Observational, prospective cohort	Paclitaxel, carboplatin	None	Paclitaxel-carboplatin chemotherapy impaired parasympathetic heart innervation and autonomic responses.
9	Jerian	Ovarian cancer patients	2	Case series	Paclitaxel	None	Paclitaxel precipitated severe autonomic neuropathy with incapacitating cardiovascular symptoms.
10	Lin	Breast cancer patients	34	Observational prospective	Anthracyclines ± taxane	None	Adjuvant chemotherapy impaired cardiovascular responses and reduced exercise tolerance during treatment.
12	Rodrigues	Anthracycline-related HFrEF patients	16	Observational, cross-sectional	Anthracycline-based	None	Anthracycline-related HFrEF showed sympathetic overactivity and reduced exercise capacity compared with controls.
13	Guimarães	Breast cancer patients	20	Case series	Anthracyclines ± trastuzumab	None	Nuclear imaging showed early cardiac sympathetic hyperactivity during treatment.
14	Kobayashi	Pediatric hematologic cancer patients	38	Observational, retrospective cohort	Anthracyclines and/or vincristine	None	Pediatric chemotherapy reduced HRV versus controls.
15	Caru	Pediatric ALL survivors	203	Observational, cross-sectional	Doxorubicin-based	Dexrazoxane	Childhood ALL survivors showed autonomic dysfunction; dexrazoxane attenuated doxorubicin-associated effects.
16	Nousiainen	Adult lymphoma patients	27	Observational, prospective cohort	Doxorubicin-based	None	Doxorubicin-induced left ventricular dysfunction was associated with an early change in sympathovagal balance towards sympathetic predominance
17	Tjeerdsma	Asymptomatic breast cancer patients	52	Observational, prospective cohort	Anthracycline-based	None	Autonomic abnormalities preceded echocardiographic changes during early anthracycline cardiotoxicity.
19	Hemu	Broad oncology patients	622	Observational, retrospective cohort	Not specified	None	Sinus tachycardia in cancer patients is associated with higher cardiovascular events and mortality.
20	Ceren	Breast cancer patients	136	Observational, cross-sectional	Not specified	None	Impaired HRV predicted worse cardiovascular risk profiles including increased occurrence of atrial fibrillation.
21	Luna-Alcala	Breast cancer patients	50	Observational, prospective cohort	Anthracyclines and trastuzumab	None	Reduced HRV predicted early cardiotoxicity before echocardiographic left ventricular dysfunction.
22	Mostarda	Breast cancer patients	18	Randomized controlled trial	Not specified	Exercise	Short-term combined exercise training improved cardiorespiratory fitness and autonomic modulation in breast cancer patients.
26	Inbara	Breast cancer patients	59	Randomized controlled trial	Anthracycline-based	Yoga	Yoga therapy showed protective effects on ANS functioning as measured by resting heart rate and HRV.
27	Park	Broad oncology patients	28	Randomized controlled trial	Not specified	Mindfulness therapy	Mindfulness therapy improved HRV, reduced distress, anger, and sleep disturbance and increased quality of life.
28	Louis	Healthy adults	40	Randomized controlled trial	None	Cryotherapy	Whole-body cryotherapy acutely increased parasympathetic activity but effects decreased with habituation.
32	Hohneck	Broad oncology patients	52	Randomized cross-over design	Not specified	Sound therapy	A single sound intervention improved cardiovascular parameters commonly associated with increased stress, including HRV (in the short term).
34	Low	Patients with neurogenic orthostatic hypotension	162	Randomized controlled trial	None	Midodrine	Midodrine significantly improved standing blood pressure and orthostatic symptoms versus placebo.
35	Irizarry-Caro	Oncology patients with heart failure	85	Observational, retrospective cohort	Not specified	Midodrine	Midodrine use in patients with cancer and heart failure showed no major adverse effects, worse cardiovascular outcomes, or increased risk of mortality.
36	Kaufmann	Patients with neurogenic orthostatic hypotension	162	Randomized controlled trial	None	Droxidopa	Droxidopa improved orthostatic symptoms and standing blood pressure compared with placebo.

This table summarizes representative human studies evaluating clinical manifestations, physiologic markers, and therapeutic interventions related to cardiovascular autonomic dysfunction in patients receiving cancer therapy. Included studies encompass observational cohorts, case series, and randomized controlled trials assessing orthostatic hypotension, sinus tachycardia, heart rate variability, and autonomic tone across diverse oncology populations, as well as selected intervention trials involving symptom-directed management. Sample size (n) reflects the final analyzed cohort when available. ALL: acute lymphoblastic leukemia; ANS: autonomic nervous system; HFrEF: heart failure with reduced ejection fraction; HRV: heart rate variability; n: number.

## Literature Search Strategy

A narrative literature search was performed using PubMed/MEDLINE and Embase to identify studies addressing cardiovascular autonomic dysfunction in patients with cancer and patients receiving cancer-directed therapy. Initial search terms included combinations of “chemotherapy,” “autonomic dysfunction,” “heart rate variability,” “orthostatic hypotension,” “sinus tachycardia,” “exercise,” “mindfulness,” “cryotherapy,” and “pharmacologic management.” Additional relevant studies were identified from the reference lists of initially selected articles. Studies were selected based on relevance to cardiovascular autonomic manifestations, proposed mechanisms, or therapeutic strategies in cancer therapy-associated autonomic dysfunction. Priority was given to human studies and oncology-specific evidence when available. When oncology-specific evidence was limited, selected non-oncology studies were included if they addressed autonomic physiology or symptom-directed management relevant to the review topic. The available evidence was interpreted qualitatively, with attention to study design, relevance to oncology populations, consistency of findings, and clinical applicability.

## Scope of the Problem, Mechanisms, and Offending Agents

Cardiovascular autonomic dysfunction is increasingly recognized in patients with cancer, particularly in advanced disease. When investigated systematically, autonomic dysfunction is highly prevalent, with up to 80% of patients demonstrating definite or severe impairment in prospective cohorts. This dysfunction is clinically significant and has been associated with increased symptom burden and reduced survival [6]. The pathophysiology is multifactorial, reflecting the combined effects of malignancy itself, cancer-directed therapies, and coexisting comorbidities. Proposed contributors include systemic inflammation, physical deconditioning, paraneoplastic processes, and treatment-related neurotoxicity. Several classes of cancer-directed therapies have been implicated, including platinum-based agents and taxanes, vinca alkaloids, anthracyclines, proteasome inhibitors, immune checkpoint inhibitors (ICIs), and radiation therapy. These exposures contribute through overlapping mechanisms such as direct neuronal injury, mitochondrial dysfunction, oxidative stress, and immune-mediated damage. A more detailed discussion of the underlying mechanisms of chemotherapy-associated autonomic dysfunction is provided in a prior review [5]. The common offending agents, their estimated burden, and proposed mechanisms are summarized in Table 2.

**Table 2:** Offending Agents, Burden (Scope of the Problem), and Mechanisms in Cardiovascular Autonomic Dysfunction

	Examples	Estimated burden	Proposed mechanisms
Platinum agents and taxanes	Cisplatin, carboplatin, paclitaxel, docetaxel	Reported in up to ∼20% of cases	Mitochondrial dysfunction, oxidative stress, impaired axonal transport, small fiber/autonomic nerve injury
Vinca alkaloids	Vincristine, vinblastine	Well-described autonomic involvement with variable prevalence	Microtubule disruption leading to axonal degeneration of autonomic fibers
Anthracyclines	Doxorubicin	Higher prevalence with cumulative dosing; reported up to ∼80% in select cohorts	Oxidative stress, mitochondrial injury, autonomic imbalance affecting cardiac regulation
Proteasome inhibitors	Bortezomib, carfilzomib	Recognized but variable prevalence	Mitochondrial dysfunction, endoplasmic reticulum stress, neurotoxicity
Immune checkpoint inhibitors	Nivolumab, pembrolizumab, ipilimumab	Rare but clinically significant	Immune-mediated neuropathy, ganglionitis, inflammatory injury
Radiation therapy	Thoracic or neck radiation	Dose- and field-dependent; often delayed	Fibrosis, vascular injury, baroreceptor and autonomic pathway damage
Cancer itself	Advanced solid tumors, metastatic disease	Detected in a majority of patients with advanced disease when systematically assessed (up to 80%)	Systemic inflammation, oxidative stress, cachexia, metabolic derangements, paraneoplastic effects
Comorbid conditions	Diabetes mellitus, malnutrition, deconditioning	Common contributors that amplify risk	Autonomic neuropathy, metabolic stress, reduced physiologic reserve

This table summarizes commonly implicated cancer-related factors and therapies with proposed mechanisms of autonomic injury, based on previously published studies.

## Manifestations of Chemotherapy-Induced Cardiovascular Autonomic Dysfunction

### Orthostatic hypotension

Orthostatic hypotension represents a clinically important yet underrecognized manifestation of chemotherapy-induced cardiovascular autonomic dysfunction. Impaired autonomic control of vascular tone and heart rate may disrupt normal baroreflex responses to postural change, resulting in inadequate peripheral vasoconstriction and insufficient chronotropic compensation upon standing. Clinically, this may present as dizziness, presyncope, syncope, fatigue, or exercise intolerance, with meaningful implications for functional status and quality of life [7]. Several chemotherapeutic agents with known neurotoxic properties, particularly taxanes and platinum-based therapies, have been associated with autonomic neuropathy affecting sympathetic efferent pathways. In this setting, orthostatic hypotension may occur independently of hypovolemia, anemia, or overt cardiac dysfunction, supporting a primary autonomic mechanism rather than a nonspecific consequence of systemic illness [8, 9]. Recognition of orthostatic hypotension in patients receiving chemotherapy is clinically relevant, as symptoms may be incorrectly attributed to deconditioning, dehydration, or treatment-related fatigue. Failure to identify an autonomic contribution may delay targeted interventions and increase the risk of falls, treatment interruptions, or dose reductions. Given the growing population of cancer survivors and the expanding use of neurotoxic and immune-modulating therapies, heightened awareness of orthostatic hypotension as a manifestation of chemotherapy-associated autonomic dysfunction is warranted.

### Sinus tachycardia

Sinus tachycardia, which may be persistent or disproportionate to physiologic demand, is frequently observed in patients with cancer. This finding may reflect autonomic dysregulation related to both malignancy and its treatment [10, 11]. In published studies, affected patients demonstrate elevated resting heart rates, abnormal chronotropic responses, or exaggerated heart rate increases with minimal exertion, most commonly described in the setting of anthracycline-based chemotherapy. Autonomic assessments in these patients frequently reveal sympathetic predominance accompanied by parasympathetic withdrawal, providing a mechanistic framework for inappropriate sinus tachycardia [12–17]. Importantly, autonomic effects appear to vary by therapeutic class. While most studies demonstrate increased sympathetic activity, ICI-associated autonomic syndromes may exhibit alternative patterns, including sympathetic dysfunction with relative parasympathetic predominance [18]. Clinically, persistent resting sinus tachycardia may contribute to palpitations, dyspnea, exercise intolerance, and fatigue, and it may identify patients at higher cardiovascular risk. In observational cancer cohorts, recurrent electrocardiogram (ECG)-confirmed sinus tachycardia around the time of cancer treatment has been associated with increased adverse cardiovascular outcomes, including heart failure, cardiac arrhythmias, and higher long-term mortality [19, 20].

### Reduced heart rate variability

Reduced heart rate variability (HRV) represents the most consistently reported marker of chemotherapy-associated cardiovascular autonomic dysfunction. HRV refers to beat-to-beat variation in heart rate and serves as a noninvasive marker of autonomic modulation of sinus node activity. In general, higher HRV reflects greater autonomic flexibility, whereas reduced HRV may suggest impaired autonomic regulation [14]. Across diverse cancer populations and treatment regimens, consistent reductions have been observed using both simple and more advanced HRV measurements. These abnormalities suggest disruption of normal beat-to-beat autonomic control of heart rate rather than isolated changes in resting heart rate alone. HRV reductions have been described in association with multiple chemotherapeutic agents, with the strongest and most consistent evidence derived from studies of anthracycline-based regimens [14–17]. Similar patterns have also been reported with other therapies, including vinca alkaloids and monoclonal antibodies such as trastuzumab, supporting the concept that autonomic impairment may occur across distinct classes of cancer treatment [13]. Importantly, HRV abnormalities may be detectable in the absence of overt structural heart disease or symptomatic heart failure, raising the possibility that autonomic dysfunction may represent an early or subclinical component of the cardiotoxicity spectrum. In this context, reduced HRV may serve not only as a physiologic marker of autonomic injury but also as a potential indicator of increased cardiovascular vulnerability during and after cancer therapy, as changes in HRV have been shown to precede echocardiographic evidence of cardiotoxicity and to reflect autonomic imbalance separate from overt structural dysfunction [21]. However, while these observations suggest a clinically important association, a direct causal relationship has not been established.

## Therapeutic Strategies for Cardiovascular Autonomic Dysfunction

### Structured exercise programs

Exercise-based training represents a promising nonpharmacologic intervention for chemotherapy-associated cardiovascular autonomic dysfunction. In oncology populations, structured exercise programs incorporating aerobic activity, with or without resistance training, have been associated with favorable changes in autonomic markers, including reductions in resting heart rate and improvements in HRV. In a human intervention study of breast cancer patients receiving adjuvant therapy, a short-term supervised combined exercise program led to significant improvements in autonomic modulation, as reflected by HRV measures, compared with usual care [22]. Consistent with these findings, a recent systematic review and meta-analysis reported overall improvements in autonomic indices among patients participating in structured exercise programs across diverse cancer populations and treatment regimens [23]. In clinical practice, exercise guidance from the American College of Sports Medicine International Multidisciplinary Roundtable recommends that cancer survivors avoid inactivity and participate in regular aerobic and resistance training when feasible. Suggested exercises include walking, cycling, or similar moderate-intensity activity for approximately 30 min per session at least three times per week, while resistance training may include light weights, resistance bands, bodyweight exercises, machines, or circuit-based training targeting major muscle groups at least two times per week [24]. Collectively, the available data support exercise as a robust therapeutic strategy, providing reproducible improvements in autonomic regulation alongside well-established benefits in functional capacity, fatigue, and quality of life. Orthostatic or tilt-table training has also been explored as a nonpharmacologic strategy for recurrent orthostatic intolerance and reflex syncope; however, available evidence remains limited, mixed, and largely derived from non-oncology populations, with no established role in chemotherapy-associated cardiovascular autonomic dysfunction [25].

### Relaxation techniques and mindfulness

Relaxation and mindfulness-based interventions have also been investigated as nonpharmacologic strategies to favorably influence autonomic regulation through enhancement of parasympathetic activity and attenuation of sympathetic tone. Yoga, a mind-body practice combining physical postures, controlled breathing, meditation, and relaxation, has been explored in oncology as a strategy to modulate autonomic function during cancer treatment. In a randomized controlled trial of breast cancer patients receiving anthracycline-based chemotherapy, an 18-week integrated yoga therapy program was associated with more favorable profiles of resting heart rate and HRV after chemotherapy compared with usual care, suggesting preservation of parasympathetic modulation and attenuation of sympathetic predominance with yoga participation [26]. Beyond physiologic effects, mindfulness-based interventions may also confer meaningful symptomatic benefit in oncology populations. In a human interventional study incorporating mindfulness-based supportive therapy, participation was associated with improvements in HRV alongside reductions in perceived stress and improvements in quality of life, suggesting that autonomic modulation and patient-reported outcomes may improve in parallel [27]. Taken together, these findings support relaxation and mindfulness-based approaches as low-risk adjunctive therapies with potential benefit across both autonomic regulation and symptom burden in patients undergoing cancer treatment.

### Cryotherapy and other exploratory interventions

Several emerging nonpharmacologic interventions have been explored as potential strategies to influence autonomic tone, reduce symptom burden, or improve physiologic recovery. Cryotherapy has received attention in this context because cold exposure has measurable effects on cardiovascular physiology. Most commonly delivered as whole-body cryotherapy (brief exposure to very cold air) or cold-water immersion, cryotherapy has been studied as a physiologic stimulus that can acutely shift cardiac autonomic control. Human experimental work shows that cold exposure can increase vagal modulation and improve HRV after exposure, findings consistent with enhanced parasympathetic activity. In a controlled, time- and dose-response study, whole-body cryotherapy altered autonomic indices and was accompanied by changes in circulating catecholamines and HRV, supporting a measurable autonomic effect. However, repeated daily exposures were associated with a diminished autonomic response, suggesting physiologic habituation [28]. More recently, a meta-analysis evaluating cold exposure modalities, including cold-water immersion and cryostimulation, concluded that these interventions generally enhance parasympathetic nervous activity, although results vary by protocol and population [29]. Optimal cryotherapy parameters for chemotherapy-associated cardiovascular autonomic dysfunction have not been established. Whole-body cryotherapy protocols studied for autonomic or cardiovascular effects typically involve brief exposure to very cold air in specialized chambers for approximately 2 to 3 min, with reported temperatures commonly ranging from approximately −60 to −110 °C, although protocols vary in frequency and number of sessions [28, 29]. Other exploratory approaches include flotation-restricted environmental stimulation therapy (Flotation-REST), also known as sensory deprivation therapy, Alpha-STIM, and acoustic vibration or sound therapies. Flotation-REST involves reduced sensory stimulation while floating in a controlled environment and has been studied for acute physiologic relaxation and autonomic effects [30]. Alpha-STIM is a cranial electrotherapy stimulation device that delivers low-intensity electrical stimulation; although it has been studied in women receiving chemotherapy for early-stage breast cancer, the trial focused on symptoms such as anxiety, depression, fatigue, pain, and sleep disturbance rather than cardiovascular autonomic endpoints and did not demonstrate significant benefit compared with sham stimulation [31]. Acoustic vibration or sound therapies use auditory or vibration-based stimulation as sensory-based interventions and have been evaluated in cancer patients for short-term effects on cardiovascular parameters, including HRV [32]. Overall, evidence supporting these approaches for chemotherapy-associated cardiovascular autonomic dysfunction remains sparse, heterogeneous, and largely indirect. Therefore, these therapies should be considered exploratory rather than established interventions in this population.

### Pharmacologic management

Pharmacologic therapy for cancer therapy-associated autonomic dysfunction is best framed as symptom-directed, most often for orthostatic hypotension and orthostatic intolerance. A practical approach, consistent with the framework outlined in the JACC State-of-the-Art Review by Freeman et al, is to first optimize reversible contributors and nonpharmacologic measures, then use medications when symptoms persist or functional limitation remains substantial. When drug therapy is required, agents generally fall into two categories: therapies that expand effective circulating volume and therapies that augment peripheral vasoconstriction [33]. For orthostatic hypotension, the most commonly used first-line pressor in clinical practice is midodrine, an oral alpha-1 agonist that increases peripheral vascular tone and improves orthostatic blood pressure and symptoms in randomized trials of neurogenic orthostatic hypotension [33, 34]. In cardio-oncology, its use can be complicated by coexisting heart failure, baseline hypertension, and drug interactions, and patients require counseling regarding supine hypertension. Real-world data in patients with cancer and concurrent heart failure suggest that midodrine is commonly used for orthostatic hypotension and was not associated with major short-term safety signals in that cohort, although larger prospective oncology-specific trials remain needed [35]. When symptoms persist or midodrine is not tolerated, droxidopa, a norepinephrine precursor, is another option with randomized trial evidence for symptomatic neurogenic orthostatic hypotension and a similar need to monitor for supine hypertension and headache [36]. Fludrocortisone can increase intravascular volume and may improve orthostatic symptoms, but in cardio-oncology its role is often constrained by edema, hypokalemia, and potential heart failure decompensation, particularly in patients with anthracycline-related left ventricle (LV) dysfunction or cancer therapy–related cardiomyopathy [5, 6, 37]. Beyond symptomatic therapy, limited evidence suggests that cardioprotective agents may mitigate chemotherapy-associated autonomic injury. In long-term survivors of childhood acute lymphoblastic leukemia (ALL) treated with anthracyclines, dexrazoxane exposure was associated with more preserved autonomic function, as reflected by HRV measures, supporting a potential role for upstream prevention of autonomic toxicity [15]. Finally, for patients in whom autonomic dysfunction presents predominantly as tachycardia with symptom burden (palpitations, exercise intolerance) rather than hypotension, rate-control strategies such as beta-blockers may be considered on a case-by-case basis. Although beta-blockers are used in related autonomic syndromes characterized by excessive sinus tachycardia, their effect in chemotherapy-associated cardiovascular autonomic dysfunction is not well established. In this population, beta-blockers may reduce palpitations or excessive tachycardic responses in selected patients, but may also blunt compensatory tachycardia or worsen fatigue, bradycardia, hypotension, or orthostatic intolerance [38]. Therefore, treatment should be individualized with careful attention to blood pressure, volume status, and coexisting cardiotoxicity.

## Summary and Conclusions

Chemotherapy-associated cardiovascular autonomic dysfunction represents an underrecognized but clinically meaningful component of cancer therapy–related cardiotoxicity. Evidence across diverse cancer populations demonstrates that disruption of autonomic regulation may manifest as orthostatic hypotension, sinus tachycardia, and reduced HRV, with important implications for symptom burden, functional capacity, and cardiovascular risk. These abnormalities may occur independently of overt structural heart disease, suggesting that autonomic dysfunction may represent an early or subclinical marker of cardiovascular vulnerability; however, a direct causal relationship with subsequent cardiotoxicity has not been established.

Importantly, emerging data indicate that autonomic dysfunction in this setting may be modifiable, although the strength of evidence varies substantially by intervention. Structured exercise programs have the strongest and most direct oncology-specific evidence, with studies demonstrating improvements in autonomic indices as well as functional capacity, fatigue, and quality of life. Relaxation and mindfulness-based interventions, including yoga and supportive mindfulness therapies, appear to offer low-risk adjunctive benefits, but the evidence base is smaller and less consistent. Cryotherapy and other emerging interventions remain the least established, with much of the available evidence derived from healthy or non-oncology populations. Pharmacologic therapies play a symptom-directed role, particularly for orthostatic hypotension, but their use in cardio-oncology populations is often constrained by comorbid heart failure, blood pressure considerations, and limited disease-specific evidence.

As the population of cancer survivors continues to grow, greater recognition of cardiovascular autonomic dysfunction is warranted. Although standardized diagnostic pathways have not yet been established, the initial evaluation should focus on the patient’s main symptoms, orthostatic vital signs, ECG findings, and medication review. Importantly, clinicians should not automatically attribute these symptoms to cancer therapy-related autonomic dysfunction without first evaluating for other common causes in oncology patients, including the malignancy itself, infection, anemia, volume depletion, or deconditioning. HRV may provide supportive information as a noninvasive measure of cardiac autonomic function. One conventional approach is paced deep breathing, in which beat-to-beat R-R interval changes are measured during inspiration and expiration; this respiratory variation reflects cardiovagal function and can be expressed as the expiratory-to-inspiratory ratio [39]. Longer-duration ambulatory ECG monitoring can also be analyzed with appropriate software to generate HRV metrics from R-R interval data [40]. However, standardized outpatient protocols and clinically validated thresholds for chemotherapy-associated cardiovascular autonomic dysfunction have not been established. Ideally, HRV assessment will become a more accessible and standardized tool for evaluating autonomic function in cardio-oncology patients. Referral to cardio-oncology, cardiology, or autonomic specialists should be considered for patients with recurrent syncope, severe or unexplained orthostatic symptoms, persistent resting tachycardia, or symptoms that do not improve after reversible contributors have been addressed.

Several limitations should be considered when interpreting the available literature. Many studies are limited by small sample sizes, heterogeneous cancer populations, variable treatment exposures, and differences in baseline cardiovascular risk. Autonomic assessment methods also vary substantially across studies, including differences in orthostatic vital sign protocols, HRV acquisition methods, HRV metrics reported, and timing of assessment relative to cancer therapy. In addition, outcome definitions are not standardized, making it difficult to compare the incidence, severity, and clinical significance of autonomic dysfunction across cohorts. Much of the evidence is observational, and several interventions, particularly cryotherapy and other emerging approaches, rely partly on data extrapolated from healthy or non-oncology populations. These limitations restrict the ability to define diagnostic thresholds, establish prognosis, or determine which interventions should be routinely recommended in cardio-oncology practice.

Future research should focus on defining standardized assessment strategies and determining how targeted interventions can be integrated into cardio-oncology care pathways. Addressing this overlooked dimension of cardiotoxicity has the potential to improve both quality of life and long-term cardiovascular outcomes for patients undergoing and surviving cancer therapy.

## Data Availability

No new data were generated or analyzed for this research. Data sharing is not applicable.

## Abbreviations

ALL: acute lymphoblastic leukemia
ANS: autonomic nervous system
ECG: electrocardiogram
ESC: European Society of Cardiology
HFrEF: heart failure with reduced ejection fraction
HRV: heart rate variability
ICI: immune checkpoint inhibitor
JACC: Journal of the American College of Cardiology
LV: left ventricle
